# P-1351. Fosmanogepix Expanded Access Experience in Patients With Fusarium Infections

**DOI:** 10.1093/ofid/ofaf695.1539

**Published:** 2026-01-11

**Authors:** Sanjeet S Dadwal, Aliyah Baluch, Jana Dickter, Jessica R Newman, M Hong Nguyen, Rachel Weihe, Joy E Gibson, Jannik Stemler, Haran Schlamm, Luis Ostrosky-Zeichner

**Affiliations:** City of Hope National Medical Center, Duarte, CA; Moffitt Cancer Center, Tampa, Florida; City of Hope National Medical Center, Duarte, CA; University of Kansas Medical Center, Fairway, Kansas; University of Pittsburgh Medical Center, Pittsburgh, Pennsylvania; University of Kansas Medical Center, Fairway, Kansas; Children’s Hospital Los Angeles, Los Angeles, California; University of Cologne, Faculty of Medicine and University Hospital Cologne, Department I for Internal Medicine, Excellence Center for Medical Mycology (ECMM), Cologne, NRW, Germany; University of Cologne, Faculty of Medicine and University Hospital Cologne, Institute of Translational Research, Cologne Excellence Cluster on Cellular Stress Responses in Aging-Associated Diseases (CECAD), Cologne, NRW, Germany, Cologne, Nordrhein-Westfalen, Germany; HTS Pharma, San Diego, California; University of Texas Health Science Center, Houston, Texas

## Abstract

**Background:**

Fosmanogepix (FMGX; prodrug, active moiety manogepix [MGX]) is the first member of the “gepix” antifungal class. FMGX inhibits Gwt1, depleting GPI-anchor proteins important for fungal cell wall integrity; it shows consistent in vitro activity against *Fusarium* spp and wide tissue distribution. FMGX is available via expanded access (EA) for patients with no alternative treatment options (NCT06433128).

**Table 1.**
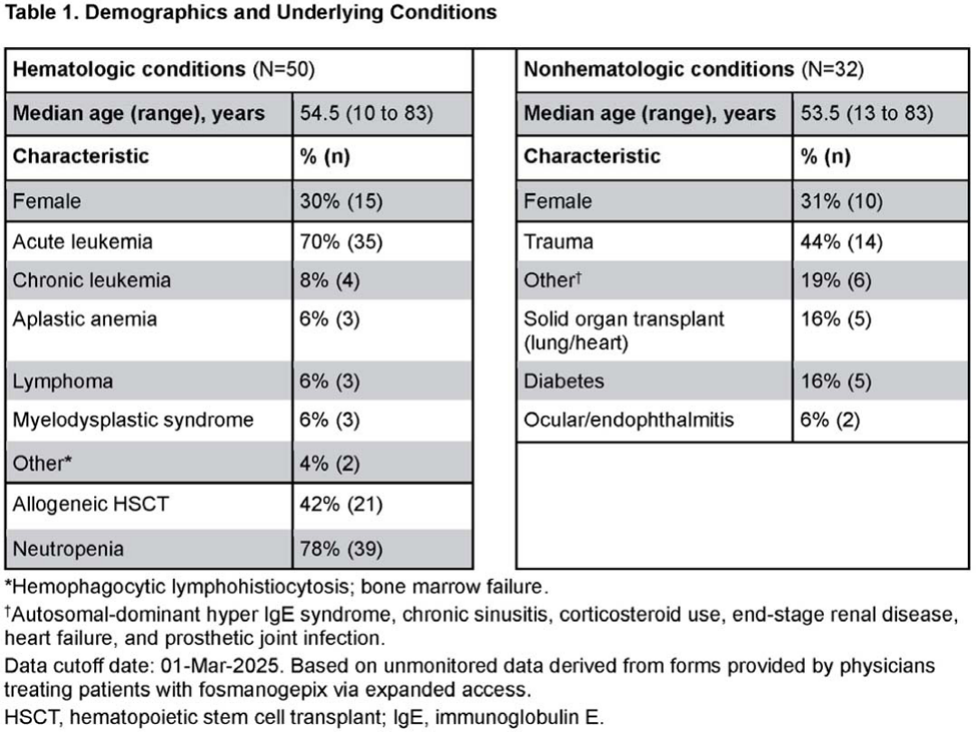
Demographics and Underlying Conditions *Hemophagocytic lymphohistiocytosis; bone marrow failure. †Autosomal-dominant hyper IgE syndrome, chronic sinusitis, corticosteroid use, end-stage renal disease, heart failure, and prosthetic joint infection. Data cutoff date: 01-Mar-2025. Based on unmonitored data derived from forms provided by physicians treating patients with fosmanogepix via expanded access. HSCT, hematopoietic stem cell transplant; IgE, immunoglobulin E.

**Table 2.**
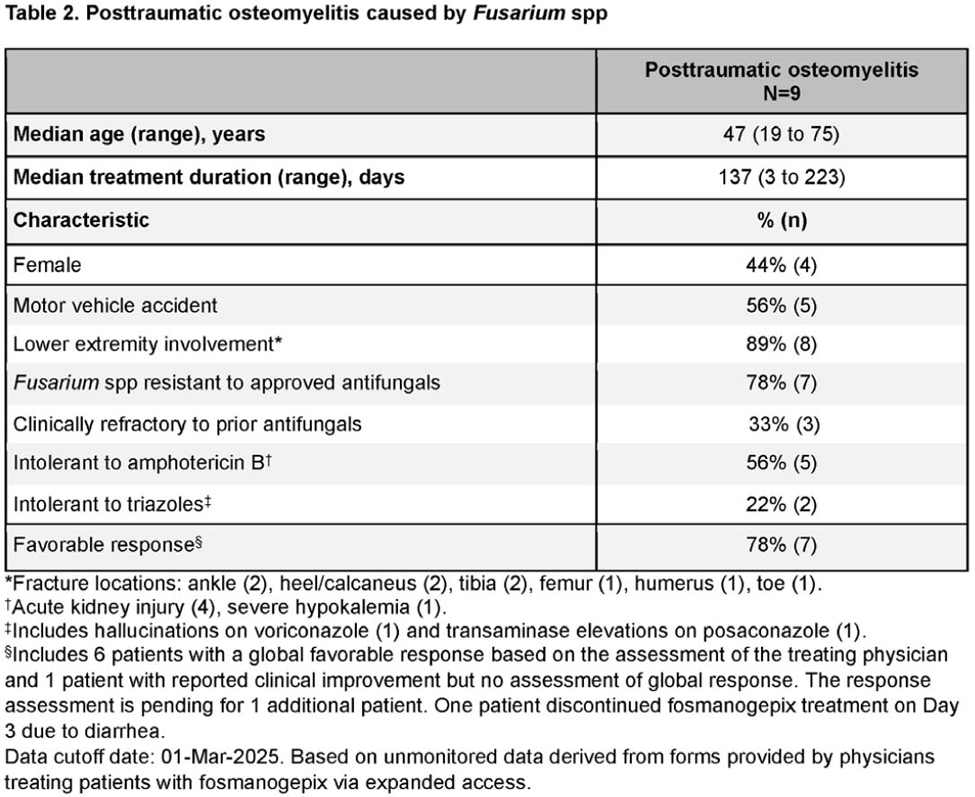
Posttraumatic osteomyelitis caused by Fusarium spp *Fracture locations: ankle (2), heel/calcaneus (2), tibia (2), femur (1), humerus (1), toe (1). †Acute kidney injury (4), severe hypokalemia (1). ‡Includes hallucinations on voriconazole (1) and transaminase elevations on posaconazole (1). §Includes 6 patients with a global favorable response based on the assessment of the treating physician and 1 patient with reported clinical improvement but no assessment of global response. The response assessment is pending for 1 additional patient. One patient discontinued fosmanogepix treatment on Day 3 due to diarrhea. Data cutoff date: 01-Mar-2025. Based on unmonitored data derived from forms provided by physicians treating patients with fosmanogepix via expanded access.

**Methods:**

Patients with documented *Fusarium* infections received FMGX via EA. Data including global response (clinical, radiological, mycological) were collected using structured forms.

**Figure 1.**
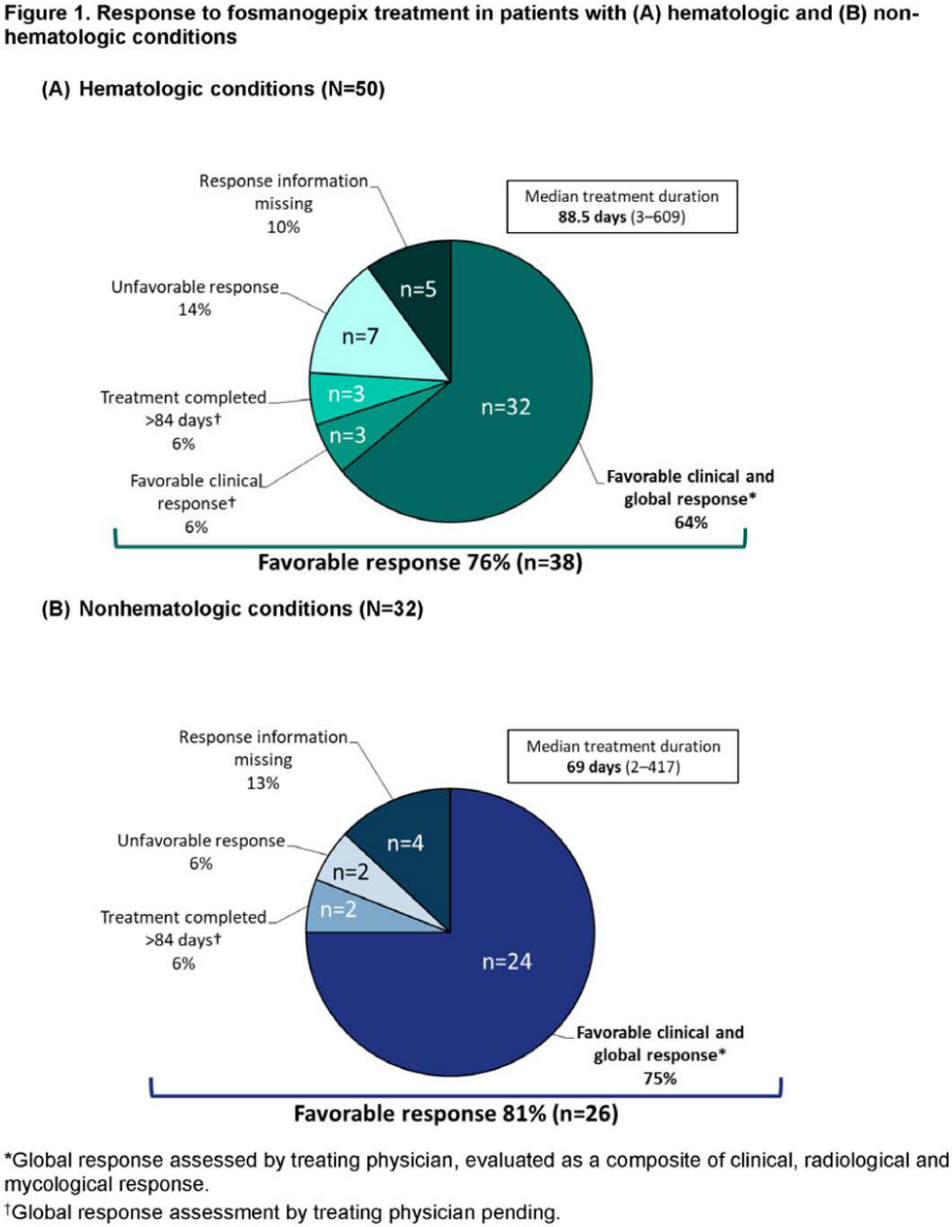
Response to fosmanogepix treatment in patients with (A) hematologic and (B) non-hematologic conditions *Global response assessed by treating physician, evaluated as a composite of clinical, radiological and mycological response. †Global response assessment by treating physician pending.

**Results:**

92 patients (34 females; median age 50.5 [range, 10–83] y) from 7 countries (92% US) completed FMGX treatment. *Fusarium solani* was most common, with high MICs for triazoles and/or amphotericin B and low MICs for MGX.

10 previously healthy people (9 female, median age 30.5 y) from the US (8) and Mexico (2) received FMGX during a fungal meningitis outbreak from iatrogenic contamination (epidural anesthesia), with 80% survival.

50 patients had hematologic conditions, mainly acute leukemia (Table 1; 21 [42%] disseminated infections, 22 bloodstream, 38 skin involvement). Response was favorable in 38 patients (76%; Fig 1A); 16 patients (32%) died (9) or transferred to hospice care (7) ≤ 6 weeks after starting FMGX. FMGX was well tolerated up to 609 (median 88.5) days.

32 patients had nonhematologic conditions (trauma [14], solid organ transplant [5], diabetes [5]; Table 1). Most infections were osteoarticular (12) and/or of skin/soft tissue (8). Overall clinical response was favorable in 26 patients (81%; Fig 1B), including 7/9 with posttraumatic osteomyelitis (OM; Table 2). No patient died ≤ 6 weeks after starting FMGX. FMGX was well tolerated up to 417 (median 69) days.

Most common adverse reactions with FMGX were gastrointestinal (eg, nausea, vomiting, diarrhea), leading to discontinuation in 3 patients.

**Conclusion:**

Based on this large prospective assessment in an EA setting, FMGX provides a potentially life-saving treatment option in difficult-to-treat *Fusarium* infections in diverse patient scenarios, including hematologic malignancies, outbreak-associated meningitis, and posttraumatic OM, and was well tolerated for long treatment durations.

**Disclosures:**

Sanjeet S. Dadwal, MD, Ansun Biopharma: Grant/Research Support|Aseptiscope, Inc.: Stocks/Bonds (Private Company)|Basilea: Advisor/Consultant|Basilea: Grant/Research Support|F2G: Grant/Research Support|Karius: Advisor/Consultant|Karius: Grant/Research Support|Karius: Honoraria|Merck: Advisor/Consultant|Pfizer: Grant/Research Support|Pulmotect: Grant/Research Support|Symbio: Grant/Research Support|Takeda: Advisor/Consultant M Hong Nguyen, MD, Basilea: Advisor/Consultant|BioMerieux: Grant/Research Support|Melinta: Grant/Research Support|Pulmocide: Advisor/Consultant|Pulmocide: Grant/Research Support Jannik Stemler, MD, AbbVie: Honoraria|Akademie für Infektionsmedizin: Honoraria|Alvea Vax: Advisor/Consultant|Basilea: Grant/Research Support|German Federal Ministry of Education and Research (BMBF): Grant/Research Support|German Society for Infectious Diseases: Travel grants|Gilead: Advisor/Consultant|Gilead: Honoraria|Hikma: Honoraria|Lilly: Honoraria|Meta-Alexander Foundation: Travel grants|Micron Research: Advisor/Consultant|Mundipharma: Honoraria|Noscendo: Grant/Research Support|Pfizer: Honoraria|Scynexis: Grant/Research Support|The Medical Faculty of the University of Cologne: Grant/Research Support Haran Schlamm, MD, Amplyx: Advisor/Consultant|Basilea: Advisor/Consultant|Pfizer: Advisor/Consultant Luis Ostrosky-Zeichner, MD, Basilea: Advisor/Consultant|Basilea: Grant/Research Support|Basilea: Honoraria|Eurofins Viracor: Advisor/Consultant|Eurofins Viracor: Grant/Research Support|Eurofins Viracor: Honoraria|F2G: Advisor/Consultant|F2G: Grant/Research Support|F2G: Honoraria|Gilead: Advisor/Consultant|Gilead: Grant/Research Support|Gilead: Honoraria|GSK: Advisor/Consultant|GSK: Grant/Research Support|GSK: Honoraria|Melinta: Advisor/Consultant|Melinta: Grant/Research Support|Melinta: Honoraria|Pfizer: Advisor/Consultant|Pfizer: Grant/Research Support|Pfizer: Honoraria|Pulmocide: Advisor/Consultant|Pulmocide: Grant/Research Support|Pulmocide: Honoraria|Scynexis: Advisor/Consultant|Scynexis: Grant/Research Support|Scynexis: Honoraria|T2 Biosystems: Advisor/Consultant|T2 Biosystems: Grant/Research Support|T2 Biosystems: Honoraria

